# Personality Traits and Potential Career Choices Among Medical Students at Sultan Qaboos University: A Cross-Sectional Study

**DOI:** 10.7759/cureus.51994

**Published:** 2024-01-10

**Authors:** Abdullah Al Sawafi, Abdullah Al Lawati, Hamed Al Sinawi

**Affiliations:** 1 Neurosurgery, College of Medicine and Health Sciences, Sultan Qaboos University, Muscat, OMN; 2 Medicine, College of Medicine and Health Sciences, Sultan Qaboos University, Muscat, OMN; 3 Psychiatry and Behavioral Sciences, Sultan Qaboos University Hospital, Muscat, OMN

**Keywords:** medical specialization, big five personality test, personality, career choices, medical students, personality traits

## Abstract

Background

Personality plays a vital role in choosing a medical specialty. Despite this, research is scarce on this subject, especially in Oman. This study aimed to investigate the correlation between the personality traits of medical students at Sultan Qaboos University (SQU), Muscat, Oman, and the specialty they wished to undertake after graduation.

Methods

This was a cross-sectional study. Two hundred and thirty-four medical students completed the Big Five personality questionnaire and were asked what specialty they would like to choose. The specialties were divided into three namely, surgical-oriented, medicine-oriented, and basic medicine. An ANOVA test was used to find any statistically significant correlation between personality traits and the desired specialty.

Results

The students who chose surgery-oriented specialties had significantly higher scores in the extraversion and openness means (P<0.05) than the students who opted for medicine-oriented specialties and basic medicine. The students who chose basic medicine had significantly higher scores in the neuroticism means (P<0.05) than the other groups.

Conclusion

The students who chose surgery-oriented specialties were more likely to be highly extraverted and open, while the medical students who chose basic medicine were more likely to have higher neuroticism levels when compared with the other groups. This study indicates that there is a correlation between personality traits and desired specialty. Further studies should be done to investigate if these personality traits remained similar after graduation.

## Introduction

Personality traits are characteristic patterns of how people think, feel, or behave in different situations [1]. People have different personality traits, and because personality traits play an important role in how people perceive the world, it is essential to factor them in when choosing their career path. This could enhance the person’s satisfaction with their career and their overall productivity [2]. Of course, personality is not the only factor that contributes to choosing a medical specialty; other factors may include gender, past experiences, family influence, peer pressure, lifestyle, income, and social status. All these factors, coupled with the fact that a medical student is rushed earlier in his career to choose a specialty, make it all the more difficult for them to choose a specialty [3-7]. So it is very difficult for medical students to choose a specialty in general, but even more difficult to choose a specialty that matches their personality traits and temperaments, so they can deeply enjoy their chosen specialty, which is vital for them to excel in their career [8-9]. This becomes a bigger problem when factoring in all the different stereotypes people have about doctors, especially the negative ones, which may deter some students from pursuing certain specialties.

Many studies show that there is a significant correlation between certain personality traits and temperaments and the chosen specialty [10-11]. For example, a Swedish study came out in 2015 that showed surgeons tended to be more conscientious and less agreeable when compared with internal medicine and physicians in primary care [12]. Another study published in 2010 indicated that surgery residents scored higher in conscientiousness, extraversion, and emotional stability but scored lower in openness when compared with the community norm [13]. However, a study posted in 2016 in the Korean Journal of Medical Education showed that although some personality traits were found more commonly in certain specialties, personal interest also played an influential role in the specialty that more open and conscientious students chose [14].

On the other hand, some studies show that there is in fact no significant correlation between personality traits and the specialty the students chose. A study published in 2019 on Polish medical students, showed that personality traits were not the predominant factor determining the specialty they chose [15]. A similar study published in 2017 conducted on residents of the Oman Medical Specialty Board (OMSB) using the Eysenck Personality Questionnaire-Revised to investigate any correlations between personality traits and specialty chosen by the residents showed that respondents from surgical specialties scored higher in psychoticism while those of psychiatry scored the lowest in neuroticism in young Omani doctors [16]. However, because the target group was residents, not medical students, their answers may have been impacted by their experiences with the different specialties during their training.

There is still no study done to investigate possible correlations between personality traits and the specialty chosen by the students who have not yet graduated from medical school in Oman. This study aimed to find any correlation between personality traits and the specialty chosen by the undergraduate medical students of Sultan Qaboos University (SQU), as it is the most prominent university in Oman. The results of this study will expand our knowledge on this topic and should greatly facilitate the specialty selection process of medical students for residencies, while simultaneously improving the medical students’ overall satisfaction as the specialty matches their personality traits. This information can also improve the educators' role in educating those students based on their personality types, which has been proven to enhance the education provided, hence improving the quality of medical students and, by extension, future healthcare [9].

## Materials and methods

Study design

This was a cross-sectional observational study. A web-based questionnaire prepared using Google Forms (Google Inc., Mountainview, CA) was disseminated to the participants. 

Study population and sample size

The subjects that were included in this study were phases 1, 2, and 3 of medical students who are currently studying in SQU. There were a total of 234 respondents to the questionnaire sent.

The questionnaire used and the method of data collection

We used the Big Five personality test, also known as the Five Factor Model (FFM) in this study to measure the personality traits of medical students. The FFM organizes the scores into five dimensions: extraversion, agreeableness, conscientiousness, neuroticism, and openness. The FFM is validated, publicly available, and has been widely replicated internationally [17]. Each of the earlier-mentioned dimensions shows a side of an individual’s personality. People have various levels of each dimension, painting a picture of the individual [18].

A high score in extraversion means that the individual is more likely to be social, assertive, energetic, adventurous, and enthusiastic. A high score in agreeableness means that the individual is more likely to be forgiving, straightforward, altruistic, compliant, and modest. A high score in conscientiousness means that the individual is more likely to be efficient, organized, dutiful, thorough, self-disciplined, and thorough. A high score in neuroticism means that the individual is more likely to be tense, irritable, depressed, shy, impulsive, moody, and vulnerable. A high score in openness means that the individual is more likely to be curious, imaginative, artistic, excitable, and unconventional.

The Big Five personality test is a self-reported questionnaire comprising 44 Likert scale questions. Each question comprises a short phrase like, “I see myself as someone who is reserved.” The respondents chose one of the five options provided based on how much they agreed with the phrase. The options were: 1) disagree strongly; 2) disagree a little; 3) neither agree nor disagree; 4) agree a little; or 5) agree strongly. The questions asked measure either extraversion, agreeableness, conscientiousness, neuroticism, or openness. A high score in any of these dimensions means the respondent showed a tendency to view themselves in line with that personality trait.

The SQU medical students received the Big Five Inventory (BFI) questionnaire in the form of an attachment to their email and via WhatsApp (Meta, Menlo Park, CA). They then completed the questionnaire on their smartphones or laptops, which usually takes about 15 minutes to complete. At the beginning of the questionnaire, all the relevant information about the questionnaire was displayed. It also mentioned that before doing the questionnaire, the students must first give their consent, and submission of the questionnaire itself will be taken as confirmation of the consent from the students. All the respondents were assured in writing that their participation and answers would be completely anonymous and voluntary, and the data collected would be aggregated. The respondents were also allowed to withdraw from the study at any point, and if they experienced any distress when answering any sensitive questions, proper support was available. Also, the respondents were advised not to discuss their answers with each other, as this may affect the results via peer influence. Informed consent from all the participants was collected, as mentioned earlier.

Ethical approval was granted from the Medical Research and Ethics Committee (MREC) at the College of Medicine and Health Sciences, SQU, Muscat, Oman.

Data analysis

Data analysis was done using the IBM SPSS software, version 27.0 (IBM Corp., Armonk, NY). The continuous variables were summarized as mean ± standard deviation. One-way ANOVA was used along with post hoc analysis to find any significant correlation between personality traits and certain specialties. A p-value of <0.05 was considered statistically significant. When there are statistically significant values, this proves that there is a correlation between the students’ personality traits and their chosen specialty.

For the sake of brevity, the specialties were divided into either surgical specialty, medical specialty, or basic medicine. In SQU, the medical students’ education is in the form of phases: phase 1 (pre-clinical 1), phase 2 (pre-clinical 2), and phase 3 (clinical). This is how the subjects were categorized.

## Results

Of the 234 respondents, 69 of them were pre-clinical 1 medical students (29.5%), 103 of them were pre-clinical 2 medical students (44%), and 62 of them were clinical medical students (26.5%). Of all the respondents, 108 were male (46.2%) and 126 were female (53.8%). Of the 108 males, 55 of them chose surgery-oriented specialties (50.9%), 34 chose medicine-oriented specialties (31.5%), and 19 chose basic medicine (17.6%). Of the 126 females, 42 chose surgery-oriented specialties (33.3%), 66 chose medicine-oriented specialties (52.4%), and 18 chose basic medicine (14.6%). There was a statistically significant association found between the gender and specialty chosen (P=0.005). The female students also got a statistically significant (P=0.009) higher score in the agreeableness mean (34.7) when compared to the male students’ score. A total of 114 of the respondents were 20 years of age or older (48.7%), and 120 were younger than 20 years (51.3%).

Of all the respondents, 100 chose a medicine-oriented specialty (42.7%), 97 chose a surgery-oriented specialty (41.5%), and 37 chose basic medicine (15.8%).

Table 1 presents the mean BFI scores organized into different phases: the mean score for agreeableness with clinical students (34.9) was significantly higher (P=0.043) than that of the pre-clinical 1 and pre-clinical 2 students.

**Table 1:** The mean for the personality traits from the Big Five Inventory (BFI) scores organized into phases

Phase		Mean	Standard deviation	Significance
Extraversion	Pre-clinical 1	21.1	4.1	Not significant
	Pre-clinical 2	20.8	4.4	
	Clinical	22.1	4.7	
Agreeableness	Pre-clinical 1	33.0	4.9	0.043
	Pre-clinical 2	34.3	3.8	
	Clinical	34.9	4.9	
Conscientiousness	Pre-clinical 1	30.0	5.3	Not significant
	Pre-clinical 2	30.1	5.3	
	Clinical	31.3	5.1	
Neuroticism	Pre-clinical 1	23.1	5.5	Not significant
	Pre-clinical 2	23.9	5.9	
	Clinical	24.3	6.4	
Openness	Pre-clinical 1	34.3	5.4	0.046
	Pre-clinical 2	34.4	5.5	
	Clinical	36.3	4.9	

The mean score for openness in clinical students (36.3) was also significantly higher (P=0.046) than that of pre-clinical 1 and pre-clinical 2 students.

**Figure 1:**
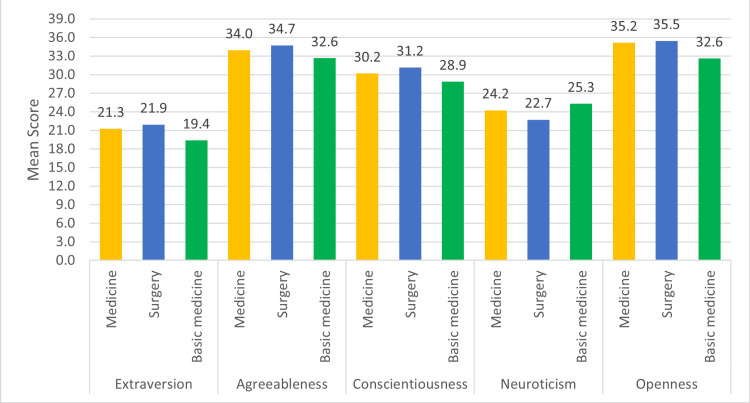
The mean score from the Big Five Inventory (BFI) scores compared with the specialty chosen

Figure 1 presents the mean BFI score divided by the FFM and compared with the specialty chosen: the mean score for extraversion with students who chose surgery-oriented specialties (21.9) was significantly higher (P=0.011) than those who chose medicine-oriented specialties and basic medicine.

It also shows that the students who chose surgery-oriented specialties (35.5) had a significantly higher score in openness (P=0.018) as well when compared to those who chose medicine-oriented specialties and basic medicine. The mean score for neuroticism (25) was significantly higher (P=0.046) in the students who chose basic medicine when compared to the students who chose surgery and medicine-oriented specialties.

## Discussion

This study further expands our knowledge of the correlation between personality and the specialty chosen by medical students. This study shows that the students who chose surgery-oriented specialties displayed a significantly higher level of extraversion when compared to the students who chose medicine-oriented specialties or basic medicine. An elevated level of extraversion means that the students who preferred surgical specialties were more likely to be outgoing, assertive, and enthusiastic when compared to the other groups. This study also shows that the students who preferred surgery-oriented specialties exhibited higher levels of openness when compared to the other groups. An elevated level of openness indicates that these students tended to be more curious and open-minded to new ideas in comparison with the students who chose medicine-oriented specialties and basic medicine. These results also show that the mean score for neuroticism was highest among the students who chose basic medicine. These data are in perfect alignment with our hypothesis and further cement that there is in fact a correlation between personality traits and the specialty chosen by the SQU medical students.

Few other studies done on this topic show similar results. A study was done on 24 male and 15 female surgical residents to find any distinct personality traits expressed more commonly in surgical residents using the Big Five personality questionnaire. The results showed that both male and female surgery residents displayed higher scores in extraversion and openness, which is in line with our results. The study also showed that surgical residents had higher conscientiousness, which was different from our results [19]. This difference can be attributed to the fact that there were no non-surgical residents in this study. Another study was conducted on 274 residents in surgery, medicine, anesthesiology, and pediatrics alongside 207 medical students to compare the personality traits exhibited by surgery residents with those exhibited by both non-surgery residents and medical students using the FMM. The findings of this study revealed that the surgery residents had higher levels of extraversion, which is in line with our findings. The findings also showed that the surgical residents scored higher means in conscientiousness and lower means in openness, which is in contrast to our findings [13]. This contrast could be a result of the fact that this test was done mainly on residents who had much more experience in the medical field and a lot more exposure to the different specialties, which more than likely had a role to play in their specialty chosen, whereas our study mainly focused on medical students who don’t have much experience in the medical field and less exposure to the different specialties.

An individual’s personality shapes the way they perceive the world, how they interact with different people, and how they react to diverse situations [20]. It also plays a role in their innovativeness and satisfaction with life [21-26]. Therefore, it is expected for personality to play a significant role in the career paths people choose to follow. As mentioned earlier, choosing a career that matches one's personality is vital to not only enjoying their professional life but also excelling in it, especially nowadays with rates of depression skyrocketing, especially among residents [27-28]. These findings also have many practical applications. By understanding the most common personality traits exhibited by students of different specialties, the medical curriculum can be specifically tailored to the student's personality traits. This would not only make the students’ learning experience much more gratifying but also make the learning much more effective, leading to better students and, eventually, better doctors and improved healthcare.

Limitations

Some limitations are present in this study. Firstly, the use of a self-reported questionnaire makes the findings prone to bias, which means the respondents might have chosen answers they feel are the morally or socially “correct” answers, which could have greatly impacted the results. In addition, personality traits do not follow a fixed metric system, so they are always prone to change due to many factors, especially age. Lastly, this was a single-center study conducted among medical students only at SQU. Further studies need to be conducted at other medical schools in Oman to generalize the findings.

## Conclusions

These results indicate that the SQU medical students who chose surgery-oriented specialties were more likely to be highly extraverted and open, while the medical students who chose basic medicine were more likely to have higher neuroticism levels when compared with the other groups. Further studies should be done on a larger sample size to minimize any erroneous trends that can be misleading. Future studies can also look for a medium other than a self-reported questionnaire to measure personality traits with less response bias to achieve more accurate results. Future studies should also investigate whether the personality traits of the SQU medical students remain similar after they graduate and undertake their chosen specialty.
